# Effectiveness of Telemedicine and Electronic Call Reminder and Health Education Trial (TECARE Trial) for Type 2 Diabetes Mellitus: A Randomized Controlled Trial

**DOI:** 10.7759/cureus.91996

**Published:** 2025-09-10

**Authors:** Vinoth Gnana Chellaiyan Devanbu, Pradakshna Porchezhian, Jennifer Britto John, Vijayalakshmi S

**Affiliations:** 1 Department of Endocrinology and Diabetes, Chettinad Superspeciality Hospital, Chettinad Academy of Research and Education, Chennai, IND; 2 Department of Endocrinology and Metabolism, All India Institute of Medical Sciences, Jodhpur, Jodhpur, IND; 3 Department of Obstetrics and Gynaecology, Chettinad Hospital and Research Institute, Chennai, IND; 4 Department of Community Medicine, Chettinad Hospital and Research Institute, Chennai, IND

**Keywords:** glycated hemoglobin, glycemic control, health communication, insulin resistance, physical activity

## Abstract

Introduction

Lifestyle management plays a vital role in the care of patients with type 2 diabetes mellitus (T2DM). Empowering individuals through education on lifestyle and behavior can be effectively supported by technology. This study aimed to evaluate the impact of telemedicine services on glycemic control, medication adherence, and insulin resistance among patients with T2DM.

Materials and methods

This two-arm, double-blinded, randomized controlled trial was conducted at a diabetes clinic in a tertiary care hospital. A total of 200 patients were enrolled and randomly assigned to either the intervention or control group. A total of 100 patients were enrolled in each of the intervention and control arms. The intervention group received weekly interactive voice calls for one year, focusing on medication adherence and lifestyle modifications, including dietary changes and physical activity. Primary outcomes included changes in HbA1c levels, medication compliance, lifestyle behaviors, and insulin resistance. An intention-to-treat analysis was performed, with a p-value of <0.05 considered statistically significant.

Results

At baseline, the mean HbA1c was 7.3% (±0.25) in the intervention group and 7.1% (±0.17) in the control group. Insulin resistance, measured by Homeostatic Model Assessment for Insulin Resistance (HOMA-IR), was 5.4 (±0.26) and 5.4 (±0.22), respectively. At 12 months, 91 participants were followed up in the intervention group, whereas 82 participants were followed up in the control group. The intervention group showed a significant reduction in HbA1c to 6.7% (±0.21), compared to 7.0% (±0.15) in the control group (p = 0.035). Fasting blood glucose and postprandial blood glucose levels also improved significantly in the intervention group (113 vs. 134 mg/dL, p = 0.046; and 142 vs. 163 mg/dL, p = 0.032, respectively). HOMA-IR was notably lower in the intervention group at 3.4 compared to 4.6 in the control group (p = 0.04).

Conclusion

Telemedicine interventions, in the form of regular health education and support, significantly improved glycemic control, lifestyle behaviors, and insulin resistance in patients with T2DM. These findings suggest that such an intervention could be further evaluated in community settings to determine its effectiveness in real-world conditions.

## Introduction

The International Diabetes Federation projects 700 million type 2 diabetes mellitus (T2DM) cases globally by 2040, up from 537 million in 2021. Low- and middle-income countries, including India, bear 75% of this burden [1-3]. In India, diabetes prevalence is 11.4% (95% CI: 10.2-12.5), with 15.3% having prediabetes [4]. Given this high disease burden, monitoring and prevention are crucial [5].

Diabetes management heavily relies on individual behaviour. A healthy lifestyle including diet, weight loss, and physical activity can reduce diabetes risk by 58% [6]. Lifestyle management, encompassing self-management support, education, and adjustments, is the key [7]. Orem et al. define self-care as an individual's responsibility for health decisions, requiring research, reflection, and action [8]. Technology can empower patients by providing lifestyle and behaviour-related information [9].

To expand healthcare access, NITI Aayog introduced telemedicine guidelines in March 2020 [10]. Given India’s rising diabetes prevalence, telemedicine can enhance diabetes care by extending services geographically [11]. Telemedicine supports self-management and clinical outcomes while offering healthcare professionals better decision-making tools via digital technology. Previous tech-based interventions, like SMS and app-based tools, had limitations due to literacy barriers and usability issues. This study, therefore, comprehensively assessed the role of telemedicine services in T2DM patients’ glycemic state and change in insulin resistance at 12 months from baseline in the intervention group among T2DM patients when compared to the control group receiving standard of care.

## Materials and methods

Study settings and participant recruitment

This double-blinded randomized controlled trial was conducted at Chettinad Hospital & Research Institute, a tertiary care hospital, in Tamil Nadu. The study included patients aged 30-65 years receiving anti-diabetes treatment.

Inclusion and exclusion criteria

The intervention group comprised patients of both genders who could speak and understand either English/ Regional (Tamil) language, having personal smart mobile phones with the WhatsApp application installed. The control group also comprised patients in the age group of 30 to 65 years, of both genders, who were willing to consult at diabetic clinics for regular follow-up every three months. Exclusion criteria for both groups were patients who were involved in any wellness programs, pregnant and lactating women, patients with a history of heart diseases, orthopedic conditions that restrict physical activity, and hearing difficulties, such as hard of hearing and difficulties in vision.

Sample size calculation and sampling

The sample size calculation for a superiority trial with the quantitative variable as an outcome measure is arrived at using the formula, n= 2 x {Z1-a/2 +Z1-b / d}2 x s2. With an expected decrease in HbA1c levels after the intervention of 1% (assumed from the previous studies [11-13]) and 80% power with a 95 % confidence interval, a statistical difference in HbA1c levels of 1.71 between the groups and a standard deviation of 1.45, the sample size was derived to be 95 and adding a dropout rate of 5%, 100 participants were enrolled in each arm, summing up a total of 200 study participants. Both groups (100 participants) had an equal number of individuals. The sampling frame was patients with diabetes, receiving treatment from the diabetes clinic of the hospital. The list of patients registered in the diabetes clinic of the hospital was used for enrolment. Registered patients received invitations to take part in the research.

Blinding, randomization and allocation concealment

The consultant physician and patients were blinded in the study. Patients who indicated interest in participating, consented and fulfilled the study's eligibility criteria underwent a randomization procedure. Block randomization techniques were used in the allocation of participants to two groups. A block of six patients was used in which three were allocated to the intervention group and three to the control group. The first patient was chosen by the lottery method to be allocated to any of the two groups. Subsequent enrolment was done using random table numbers, with odd and even numbers allocated to two groups. The SNOSE (sequentially numbered, opaque, sealed envelopes) method was used to conceal the randomization sequence, which is opened only when a participant's data is recorded. Follow-up visits for the two groups did not overlap, to ensure that the participants of the two groups did not have any interaction.

Study methods

The duration of the study was for two years from 01.09.2022 to 31.08.2024. The intervention period was for 12 months. Baseline assessments included a questionnaire on sociodemographic details, anthropometric measurements, and biochemical tests such as lipid profiles, HbA1c, and fasting/postprandial blood glucose. Insulin resistance was evaluated using the Homeostatic Model Assessment (HOMA) model, which assesses glucose-insulin dynamics. The HOMA model was used to measure insulin resistance and predict steady-state glucose and insulin concentrations during fasting for a broad range of potential combinations of insulin resistance and β-cell activity. It simulates the link between glucose and insulin dynamics. While insulin-mediated glucose synthesis via the liver regulates glucose concentrations, insulin levels are dependent on the pancreatic β-cell response to glucose concentrations. A reliable clinical and epidemiological approach for evaluating insulin resistance is the HOMA model. HOMA describes this glucose-insulin homeostasis using a set of simple nonlinear equations that are theoretically obtained. A blood sample obtained during fasting is used in the simplified approximation equation for insulin resistance. It is computed by using a constant and dividing the insulin-glucose product. An indicator of insulin resistance in the liver is the product of FPG and FPI.

Patients of the intervention arm received an automated phone call once a week, except on Sundays and festival days/ regional holidays. If the patient missed/ failed to answer the call, a repeat call was made on the same day, a maximum of three times till the patient attended the call. If the patient could not be contacted even after three attempts, the patient was contacted the next day. Patients had options to choose the language of their choice between English and Tamil. Following this, they received health education on four topics, namely diabetes and its complications, compliance to medication, lifestyle changes like physical activity, diet, and exercise, and finally health education about co-morbidities. The frequency of intervention was four weeks a month and repeated every month for twelve months.

In addition, video teleconsultation sessions were arranged with a consultant physician who was not involved in any part of the study, like participant selection, randomization, or analysis, once every month for twelve months for those in the intervention group for personalized advice. In circumstances where video teleconsultation is not possible, audio teleconsultation services were provided. The telemedicine software used provided the data of participants who attended the call and the duration of the call, which ensured the delivery of the intervention.

Control group participants attended in-person clinic visits every three months. At 12 months, all participants underwent end-line assessments, including anthropometric measurements, dietary and physical activity evaluations, drug compliance monitoring, and biochemical tests such as HbA1c and insulin resistance.

Study endpoint

The study outcomes included a reduction in HbA1c, improved medication adherence, positive lifestyle changes, and reduced insulin resistance. Medication adherence was defined as taking prescribed medication correctly in terms of dosage, frequency, and duration without missing doses. Dietary compliance was met if calorie intake stayed within 10% of the recommended value, assessed via 24-hour recall. Physical activity was considered adequate if participants walked 150 minutes per week per the WHO guidelines. Cessation of alcohol, tobacco, and betel nut use indicated compliance with behavioral changes. Glycemic outcomes included improvements in fasting blood glucose (FBG), postprandial blood glucose (PPBG), and HbA1c levels, along with changes in lipid profiles. Assessment was done by the investigator of the study to ensure blinding.

Statistical analysis

Data were analyzed with IBM SPSS Statistics for Windows, Version 23 (Released 2015; IBM Corp., Armonk, New York, United States). Quantitative variables are expressed in means and categorical variables in proportions. The primary outcome was measured to evaluate the efficacy of the intervention by comparing the study arms - decrease in HbA1c levels and secondary outcomes- adherence to management practices were measured in proportions. After checking the normality of the data, tests of significance were applied. Intention-to-treat analysis was done. Chi-square test, paired t-test, Wilcoxon rank sum test, and independent tests were used to test the hypothesis. A p-value of less than 0.05 was considered significant.

Ethical considerations

The study participants’ names were not collected in the research proforma, and informed consent was obtained. This in addition to abiding by medical research ethics, avoided bias. All stages of the study ensured the privacy and confidentiality of the participants. Institutional Ethical Committee clearance was obtained (IHEC registration number: IHEC-II/0237/22). The trial has been registered under the clinical trial registry with the number: CTRI/2024/03/064346. The project was funded by the Indian Council of Medical Research (ICMR).

## Results

**Figure 1:**
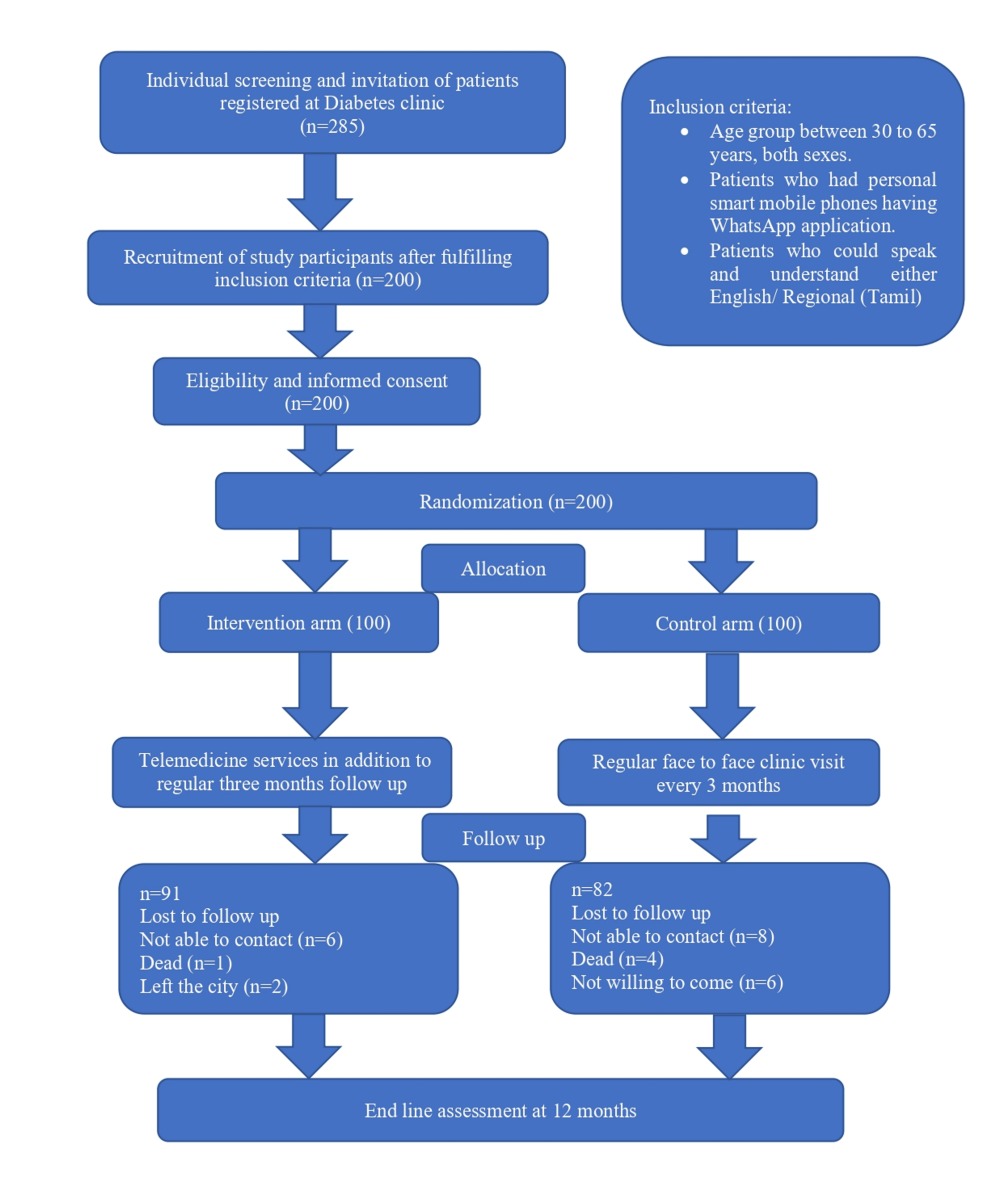
Study flowchart

A total of 200 participants were enrolled, with 100 in each group. the intervention group (Group 1) received telemedicine services in addition to routine follow-ups, while the control group (Group 2) followed standard care protocols with clinic visits every three months. About nine participants (9%) lost to follow-up in the intervention group and 18 participants (18%) lost to follow-up in the control group (Figure 1).

The mean age of participants was 47 (±8.8) years, with similar distributions across groups. Group 1 comprised 53 male participants (53%, n=53) and 47 female participants (47%, n=47), while Group 2 had 64 male participants (64%, n=64) and 36 female participants (36%, n=36). Socioeconomic and educational backgrounds were comparable between groups, with most participants (81%, n=81 in Group 1 and 64%, n=64 in Group 2) having completed high school. The majority of participants (61%, n=61 in Group 1 and 68%, n=68 in Group 2) had been diagnosed with diabetes for 2 to 5 years. Hypertension was the most common comorbidity, affecting 11% (n=11) in Group 1 and 8% (n=8) in Group 2. Smoking and alcohol consumption were reported in 2% (n=2) and 9% (n=9) of Group 1 and 4% (n=4) and 5% (n=5) of Group 2, respectively, Table 1.

**Table 1:** Study participant’s baseline characteristics (n=200). *Socioeconomic status classified by Modified BG Prasad’s Socioeconomic status scale (May 2025), chi-square statistic was used, and p>0.05 shows that there is no significant difference between the groups.

S.No.	Profile of study participants	Intervention group, Total n=100 n (%)	Control group, Total n=100 n (%)	Chi-square value	p-value
1.	Age group (in years)				
	30-45	38(38)	38(38)	1.7404	0.419
	46-59	55(55)	59(59)		
	> 60	7(7)	3(3)		
2.	Sex				
	Male	53(53)	64(64)	4.3459	0.16
	Female	47(47)	36(36)		
3.	Education				
	Primary	1(1)	19(19)	0.8969	0.746
	Middle	7(7)	6(6)		
	High School	81(81)	4(64)		
	Graduate	11(11)	11(11)		
4.	Occupation				
	Professional	0	1(1)	0.5654	0.340
	Farm/Clerk/Shop owners	84(84)	79(79)		
	Skilled/ Semiskilled	3(3)	10(10)		
	Unskilled	7(7)	6(6)		
	Unemployed/retired	4(4)	2(2)		
	Housewife	2(2)	2(2)		
5.	Socioeconomic class*				
	Class I	4(4)	0	1.8941	0.09
	Class II	19(19)	25(25)		
	Class III	34(34)	29(29)		
	Class IV	36(36)	29(29)		
	Class V	7(7)	17(17)		
6.	Place of Residence				
	Urban	89(89)	2(82)	18.2700	0.160
	Rural	11(11)	18(18)		
7.	Family type				
	Nuclear	66(66)	56(56)	0.0291	0.183
	Joint	33(33)	44(44)		
8.	Duration of diabetes				
	1 year	1(1)	0	1.9577	0.388
	2-5 years	61(61)	68(68)		
	5-10 years	38(38)	32(32)		
9.	Family history of diabetes				
	Yes	23(23)	21(21)	1.6599	0.704
	No	77(77)	79(79)		
10.	Comorbidities				
	Hypertension	11(11)	8(8)	1.6457	0.639
	Thyroid	2(2)	1(1)		
	None	87(87)	91(91)		
11.	Smoking				
	Current consumer	2(2)	4(4)	5.6662	0.36
	Past consumer	49(49)	40(40)		
	Never smoked	49(49)	56(56)		
12.	Alcohol				
	Current consumer	9(9)	5(5)	1.4519	0.118
	Past consumer	64(64)	55(55)		
	Never consumed	7(27)	40(40)		
13.	Diet history				
	Vegetarian	36(36)	30(30)	0.2068	0.367
	Non-vegetarian	64(64)	70(70)		
14.	Vegetable consumption per day				
	2 servings	8(8)	6(6)	0.0000	0.579
	1 serving	92(92)	94(94)		
15.	Fruit consumption per day				
	1 serving	22(22)	14(14)	2.0595	0.141
	none	78(78)	6(86)		
16.	Regular physical activity				
	Yes	0(30)	34(34)	2.0434	0.544
	No	70(70)	66(66)		
17.	Moderate physical activity				
	Yes	16(16)	9(9)	10.1355	0.134
	No	84(84)	91(91)		
18.	Severe physical activity				
	Yes	4(4)	5(5)	0.0768	0.733
	No	96(96)	95(95)		

Table 1 also shows the dietary and physical activity patterns of participants. A majority followed a non-vegetarian diet (64% in Group 1, 70% in Group 2), while daily vegetable (8% vs. 6%) and fruit consumption (22% vs. 14%) were low in both groups. Regular physical activity was reported by 30% in Group 1 and 34% in Group 2, with moderate activity levels being lower (16% vs. 9%). Severe physical activity was minimal in both groups (4% vs. 5%). These findings highlight the need for lifestyle interventions.

The participant's weight (in kg) varied between 41 and 78 kg in Group 1 with a mean (SD) of 64 (±12) and 58-81 kg in Group 2 with a mean (SD) of 60(±13). Mean (SD) of BMI was 24.8 (±4.4) and 23.8 (±4.7), respectively. The mean and SD of the glycemic parameters and fasting lipid profile of both the groups at baseline are tabulated in Table 2.

**Table 2:** Lab parameters of the study population at baseline (n=200). HOMA-IR: Homeostatic Model Assessment-Insulin Resistance

S. No.	Baseline values	Intervention group, n=100, Mean (±SD)	Control group, n=100, Mean (±SD)
Glycemic parameters			
1.	HbA1c (%)	7.3(±0.25)	7.1(±0.17)
2.	Fasting blood sugar (gm/dl)	150(±27)	152(±27)
3.	Postprandial blood sugar (gm/dl)	173(±38)	169(±34)
Fasting lipid profile			
1.	Total cholesterol (mg/dl)	158(±55)	160(±58)
2.	Triglycerides (mg/dl)	179(±138)	174(±154)
3.	HDL (mg/dl)	30(±11)	31(±11)
4.	LDL (mg/dl)	89(±43)	90(±40)
5.	VLDL (mg/dl)	35(±21)	33(±18)
6.	HOMA-IR	5.4(±0.26)	5.4(±0.22)

At baseline, mean glycated haemoglobin (HbA1c) levels were 7.3% (±0.25) in Group 1 and 7.1% (±0.17) in Group 2. FBG levels were 150 mg/dL (±27) and 152 mg/dL (±27), while PPBG levels were 173 mg/dL (±38) and 169 mg/dL (±34) for Group 1 and Group 2, respectively. Homeostatic Model Assessment for Insulin Resistance (HOMA-IR) was 5.4 (±0.26) in both groups at baseline. After 12 months, Group 1 showed a significant reduction in HbA1c levels to 6.7% (±0.21), while Group 2 had a smaller reduction to 7.0% (±0.15) (p=0.035). FBG decreased to 113 mg/dL (±16) in Group 1 and 134 mg/dL (±25) in Group 2 (p=0.046). PPBG levels improved to 142 mg/dL (±32) and 163 mg/dL (±36) in Groups 1 and 2, respectively (p=0.032). Insulin resistance was significantly lower in Group 1 at 3.4 (±0.29) compared to 4.6 (±0.18) in Group 2 (p=0.04). At baseline, total cholesterol was 158 mg/dL (±55) in Group 1 and 160 mg/dL (±58) in Group 2. Triglyceride levels were 179 mg/dL (±138) and 174 mg/dL (±154), while low-density lipoprotein (LDL) was 89 mg/dL (±43) and 90 mg/dL (±40) for Groups 1 and 2, respectively. High-density lipoprotein (HDL) levels were comparable at 30 mg/dL (±11) and 31 mg/dL (±11) in Group 1 and Group 2, respectively.

At 12 months, LDL levels showed a significant reduction in Group 1 (76 mg/dL ±41) compared to Group 2 (89 mg/dL ±35) (p=0.012). Other lipid parameters, including total cholesterol, triglycerides, HDL, and very low-density lipoprotein (VLDL), did not show statistically significant differences between groups (Tables 3, 4). Participants in Group 1 demonstrated notable improvements in dietary habits and physical activity. Vegetable and fruit intake increased significantly, and 70% (n=70) adhered to regular physical activity compared to 34% (n=34) in Group 2. Medication adherence was 100% (n=100) in Group 1, whereas it was lower in Group 2.

**Table 3:** Comparison of glycemic values among participants in the intervention group and control group at 12 months follow-up. An independent t-test is applied; p-value<0.05 is significant Significant p-values are indicated in bold.

S. No.	Variables	Mean (SD)		t value	Mean Difference	95% CI	p-value
		Intervention group (n=91)	Control group (n=82)				
1.	HbA1c values (%)	6.7(±0.21)	7(±0.15)	-12.32	2.140	1.13-3.261	0.035
2.	Fasting blood glucose values (g/dl)	113(±16)	134(±25)	-14.092	2.020	1.23-3.213	0.046
3.	Postprandial blood glucose values (g/dl)	142(±32)	163(±36)	-20.982	2.260	1.46-4.26	0.032
4.	Total cholesterol (mg/dl)	145(±44)	152(±53)	-9.32	-6.5	-20.47-7.24	0.352
5.	LDL (mg/dl)	76(±41)	89(±35)	-2.54	-13.85	-24.58-3.11	0.012
6.	HDL (mg/dl)	39(±18)	36(±15)	1.315	3.230	-1.613-8.07	0.190
7.	VLDL (mg/dl)	31(±19)	27(±16)	1.287	3.27	-1.73-8.27	0.199
8.	Triglycerides (mg/dl)	138(±85)	147(±84)	-0.770	-9.250	-32.95-14.45	0.442
9.	Insulin resistance	4.6(±0.18)	3.4(±0.29)	-28.911	-1.007	-1.075-0.938	0.04

**Table 4:** Mean difference in insulin resistance and lipid profile values before and after treatment among the patients in the intervention group (n=91). *Paired t-test and **Wilcoxon rank sum test were done; p-value ≤ 0.05 is considered significant Significant p-values are indicated in bold.

S. No.	Parameters	Mean (SD)	Mean difference	SD	95% CI	Test statistic	p-value
1.	Serum HbA1c (%)*						
	At baseline	7.3(±0.25)	0.2430	0.0100	0.22-0.26	24.36	0.032
	At 12 months	6.7(±0.21)					
2.	FBG (g/dl)*						
	At baseline	150(±27)	37.0	27.59	31.52-42.47	13.41	0.029
	At 12 months	113(±16)					
3.	PPBG (g/dl)*						
	At baseline	173(±38)	31.25	40.18	23.27-39.22	0.776	0.02
	At 12 months	142(±32)					
4.	Insulin resistance values*						
	At baseline	5.4(±0.26)	0.99	0.3418	0.92-1.06	29.05	0.000
	At 12 months	3.4(±0.18)					
5.	Total cholesterol (mg/dl)*						
	At baseline	158(±55)	12.22	42.31	3.82-20.61	2.88	0.213
	At 12 months	145(±44)					
6.	Triglycerides (mg/dl)**						
	At baseline	179(±138)	35.78	125.87	10.81- 60.74	2.84	0.047
	At 12 months	138(±85)					
7.	HDL (mg/dl)*						
	At baseline	30(±11)	-9.53	18.09	-13.12- -5.94	-5.26	0.234
	At 12 months	39(±18)					
8.	LDL (mg/dl)**						
	At baseline	89(±43)	4.88	16.21	1.66- 8.09	3.009	0.03
	At 12 months	76(±41)					
9.	VLDL (mg/dl)**						
	At baseline	35(±21)	13.87	34.80	6.96- 20.00	3.986	0.219
	At 12 months	31(±27)					

## Discussion

This study demonstrated that interactive voice calls (once a week) to a group of patients regarding adherence to recommended drug intake, and lifestyle modification measures such as diet, and physical exercise could significantly reduce the FBS, PPBS, HbA1c, and HOMA-IR values in the intervention group when compared to the control group. A systematic review and meta-analysis of RCTs by Hu et al. [14] reported that the clinical significance of the HbA1c level decreased in the intervention group for whom telemedicine services were provided compared to the control group who received usual treatment, which is similar to our findings. However, they did not find a reduction in BMI, which is also in line with our findings. Though there was a minimal reduction in the BMI, it was not significant.

A systematic review and meta-analysis done by Lee et al. [15] revealed that telemedicine interventions resulted in a marginally significant reduction in HbA1c levels in contrast to receiving usual care (MD: -0.55, 95% CI: -0.73 to - 0.36), which is similar to the outcomes of our study. A study done by Wu et al. [16] reported that diabetic patients who received telehealth had better glycemic control than those who received standard treatment (weighted mean difference = -0.22%; 95% confidence intervals, -0.28 to -0.15; P .001), which is similar to our study outcomes. Hussein et al. [12] and Abbas et al. [13] in their studies demonstrated that glycemic control improved in patients with T2DM who received health education with Short Message Services.

In our study, a comparison of insulin resistance between the two groups revealed that mean insulin resistance (HOMA IR) in the intervention group was found to be much lower at the end of 12 months when compared with the control group (p-value = 0.0001). A study by Motahari-Tabari et al. [17] reported that there was a reduction in insulin resistance after intervention by health education and physical activity, which is in line with our findings.

A study by Mokabel et al. [18] reported that there was a significant reduction in BMI in the intervention group following the diabetic educational programme, which included self-checks of blood sugar, foot care, exercises, and lifestyle changes. In the present study, there has been a considerable change in the lifestyle of participants in the intervention group in terms of vegetable and fruit consumption, physical activity, and smoking cessation. A study by Hu et al. [14] reported no change in BMI at the end of the intervention. Mokabel et al. [18] also reported that compliance with oral hypoglycemics improved after health education. They also reported that adherence to physical activity and dietary advice was better after intervention. Similar findings were reported in a study conducted by Pfammatter et al. [19].

Research studies have also established the fact that telehealth interventions can significantly reduce the incidence of T2DM. Ramachandran et al. [20] in their trial proved that telehealth services for lifestyle modification can significantly reduce the incidence of T2DM in the intervention group. Repeated sensitization via regular telemedicine calls, alert systems about lifestyle behavior changes, could aid in the improvement of insulin sensitivity and thereby better glycemic control.

Strengths and limitations

The study's key strengths include its robust randomized controlled trial design and double blinding, which minimized bias and enabled strong causal inference. The intervention utilized widely accessible and locally relevant technologies, such as voice calls and video consultations, making it both scalable and cost-effective, while also ensuring cultural appropriateness. By evaluating a comprehensive set of outcomes, including clinical indicators (HbA1c, FBG, PPBG, HOMA-IR), behavioral factors (diet and physical activity), and medication adherence, the study offers a holistic assessment of the intervention's effectiveness.

However, there are several limitations. The study was conducted at a single site, and the follow-up period was limited to 12 months, leaving the long-term sustainability of the observed benefits uncertain. Additionally, reliance on self-reported measures for diet and physical activity introduces the possibility of recall and social desirability biases. The inclusion criteria, which required access to a smartphone and WhatsApp, may have inadvertently excluded lower-income individuals or older adults who lack such technology, potentially limiting the generalizability of the findings.

## Conclusions

Telemedicine services, delivered through frequent and focused health education, effectively improved glycemic control and promoted healthier lifestyle behaviors among patients with T2DM. Regular call reminders contributed to better medication adherence and a significant reduction in insulin resistance. These findings highlight the potential of structured telehealth interventions in supporting chronic disease management. The results also provide a foundation for developing scalable, technology-driven support systems that could be adapted for managing other chronic conditions in diverse settings.

## References

[1] (2025). International Diabetes Federation. IDF Diabetes Atlas: Tenth edition. https://diabetesatlas.org/atlas/tenth-edition/.

[2] Shen J, Kondal D, Rubinstein A (2016). A multiethnic study of pre-diabetes and diabetes in LMIC. Glob Heart.

[3] Zimmet PZ, Magliano DJ, Herman WH, Shaw JE (2014). Diabetes: a 21st century challenge. Lancet Diabetes Endocrinol.

[4] Anjana RM, Unnikrishnan R, Deepa M (2023). Metabolic non-communicable disease health report of India: the ICMR-INDIAB national cross-sectional study (ICMR-INDIAB-17). Lancet Diabetes Endocrinol.

[5] Misra A, Khurana L (2011). Obesity-related non-communicable diseases: South Asians vs White Caucasians. Int J Obes (Lond).

[6] (2002). Reduction in the incidence of type 2 diabetes with lifestyle intervention or metformin. N Engl J Med.

[7] (2025). 4. Lifestyle management. Diabetes Care.

[8] Isik E, Fredland NM (2023). Orem’s self-care deficit nursing theory to improve children’s self-care: an integrative review. J Sch Nurs.

[9] Muralidharan S, Ranjani H, Anjana RM, Allender S, Mohan V (2017). Mobile health technology in the prevention and management of type 2 diabetes. Indian J Endocrinol Metab.

[10] Ventola CL (2014). Mobile devices and apps for health care professionals: uses and benefits. P T.

[11] (2025). Telemedicine Practice Guidelines. https://esanjeevani.mohfw.gov.in/assets/guidelines/Telemedicine_Practice_Guidelines.pdf.

[12] Hussein WI, Hasan K, Jaradat AA (2011). Effectiveness of mobile phone short message service on diabetes mellitus management; the SMS-DM study. Diabetes Res Clin Pract.

[13] Bin Abbas B, Al Fares A, Jabbari M, El Dali A, Al Orifi F (2015). Effect of mobile phone short text messages on glycemic control in type 2 diabetes. Int J Endocrinol Metab.

[14] Hu Y, Wen X, Wang F, Yang D, Liu S, Li P, Xu J (2019). Effect of telemedicine intervention on hypoglycaemia in diabetes patients: a systematic review and meta-analysis of randomised controlled trials. J Telemed Telecare.

[15] Lee PA, Greenfield G, Pappas Y (2018). The impact of telehealth remote patient monitoring on glycemic control in type 2 diabetes: a systematic review and meta-analysis of systematic reviews of randomised controlled trials. BMC Health Serv Res.

[16] Wu C, Wu Z, Yang L (2018). Evaluation of the clinical outcomes of telehealth for managing diabetes: a PRISMA-compliant meta-analysis. Medicine (Baltimore).

[17] Motahari-Tabari N, Ahmad Shirvani M, Shirzad-E-Ahoodashty M, Yousefi-Abdolmaleki E, Teimourzadeh M (2014). The effect of 8 weeks aerobic exercise on insulin resistance in type 2 diabetes: a randomized clinical trial. Glob J Health Sci.

[18] Mokabel FM, Aboulazm SF, Hassan HE, Al-Qahtani MF, Alrashedi SF, Zainuddin FA (2017). The efficacy of a diabetic educational program and predictors of compliance of patients with noninsulin-dependent (type 2) diabetes mellitus in Al-Khobar, Saudi Arabia. J Family Community Med.

[19] Pfammatter A, Spring B, Saligram N (2016). mHealth intervention to improve diabetes risk behaviors in India: a prospective, parallel group cohort study. J Med Internet Res.

[20] Ramachandran A, Snehalatha C, Ram J (2013). Effectiveness of mobile phone messaging in prevention of type 2 diabetes by lifestyle modification in men in India: a prospective, parallel-group, randomised controlled trial. Lancet Diabetes Endocrinol.

